# Activity-dependent redistribution of CaMKII in the postsynaptic compartment of hippocampal neurons

**DOI:** 10.1186/s13041-020-00594-5

**Published:** 2020-04-01

**Authors:** Jung-Hwa Tao-Cheng

**Affiliations:** NINDS Electron Microscopy Facility, National Institute of Neurological Disorders and Stroke, National Institutes of Health, Bethesda, MD 20892 USA

**Keywords:** Electron microscopy, Immunogold labeling, CaMKII, PSD, Shank

## Abstract

Calcium/calmodulin-dependent protein kinase II (CaMKII), an abundant protein in neurons, is involved in synaptic plasticity and learning. CaMKII associates with multiple proteins located at or near the postsynaptic density (PSD), and CaMKII is known to translocate from cytoplasm to PSD under excitatory conditions. The present study examined the laminar distribution of CaMKII at the PSD by immunogold labeling in dissociated hippocampal cultures under low calcium (EGTA or APV), control, and stimulated (depolarization with high K^+^ or NMDA) conditions. The patterns of CaMKII distribution are classified with particular reference to the two layers of the PSD: (1) the PSD core, a layer within ~ 30–40 nm to the postsynaptic membrane, and (2) the PSD pallium, a deeper layer beyond the PSD core, ~ 100–120 nm from the postsynaptic membrane. Under low calcium conditions, a subpopulation (40%) of synapses stood out with no CaMKII labeling at the PSD, indicating that localization of CaMKII at the PSD is sensitive to calcium levels. Under control conditions, the majority (~ 60–70%) of synapses had label for CaMKII dispersed evenly in the spine, including the PSD and the nearby cytoplasm. Upon stimulation, the majority (60–75%) of synapses had label for CaMKII concentrated at the PSD, delineating the PSD pallium from the cytoplasm. Median distance of label for CaMKII to postsynaptic membrane was higher in low calcium samples (68–77 nm), than in control (59–63 nm) and stimulated samples (49–53 nm). Thus, upon stimulation, not only more CaMKII translocated to the PSD, but they also were closer to the postsynaptic membrane. Additionally, there were two relatively infrequent labeling patterns that may represent intermediate stages of CaMKII distribution between basal and stimulated conditions: (1) one type showed label preferentially localized near the PSD core where CaMKII may be binding to NR2B, an NMDA receptor concentrated at the PSD core, and (2) the second type showed label preferentially in the PSD pallium, where CaMKII may be binding to Shank, a PSD scaffold protein located in the PSD pallium. Both of these distribution patterns may portray the initial stages of CaMKII translocation upon synaptic activation. In addition to binding to PSD proteins, the concentrated CaMKII labeling at the PSD under heightened excitatory conditions could also be formed by self-clustering of CaMKII molecules recruited to the PSD. Most importantly, these accumulated CaMKII molecules do not extend beyond the border of the PSD pallium, and are likely held in the pallium by binding to Shank under these conditions.

## Introduction

Calcium/calmodulin-dependent protein kinase II (CaMKII), an abundant protein in neurons, is involved in synaptic plasticity and learning, and can associate with many proteins at the postsynaptic density (PSD) [1, 2]. Activity-dependent translocation of CaMKII to the PSD is well-documented [3–6]. Immunogold labeling illustrated that label for CaMKII is typically evenly distributed in the spine under basal conditions, but becomes concentrated at the PSD upon stimulation. A recent review [7] further divides PSD into two layers: (1) the PSD core, a distinctive layer of dense materials ~ 30–40 nm from the postsynaptic membrane, and (2) the PSD “pallium”, a deeper layer that is contiguous with the PD core, but extending further into the cytoplasm ~ 100–120 nm from the postsynaptic membrane. This deeper layer of PSD is typically not apparent under basal conditions without special stains [8], and only becomes easily recognizable upon stimulation [7]. The present study examined the stimulation-induced redistribution of CaMKII labels with particular reference to their presence in the PSD core and the PSD pallium, and looked for clues as to how CaMKII become concentrated at the PSD upon stimulation.

Laminar distribution of CaMKII at the PSD may provide clues to CaMKII’s interactions with other PSD proteins that are localized at different layers within the PSD. The distances of CaMKII labels to the postsynaptic membrane have previously been measured from high pressure frozen and cryosubstituted brain slices [9] and from perfusion-fixed brains [10], and the two studies showed similar laminar distribution of CaMKII with a concentrated peak at ~ 40 nm, and a wide-spread range from 0 to greater than 100 nm from the postsynaptic membrane. However, to date, no distance data of CaMKII distribution under different activity states were reported. Here in dissociated hippocampal cultures where the activity state of synapses can be easily manipulated, we set out to do distance measurement under low calcium, control and stimulated conditions to illustrate the activity-related redistribution of CaMKII. The laminar distribution of CaMKII was also compared to those of other PSD proteins to examine their possible interactions under different conditions.

## Methods

### Preparation, treatment and fixation of rat dissociated hippocampal neuronal cultures

Most samples were from previously published reports [4, 5, 11–13] and reexamined here for additional analyses. Briefly, cell cultures were prepared from embryonic 20 day-old rat fetuses by papain dissociation, and then plated on glial feeder cultures, and experiments were carried out with 3 week-old cultures. Control incubation media was HEPES-based Kreb’s Ringer at pH 7.4. High K^+^ media was at 90 mM KCl, with osmolarity compensated by reducing the concentration of NaCl. N-methyl-D-aspartic acid (NMDA) media contained 50–60 μM NMDA in the control media. Culture dishes were placed on a floating platform in a water bath maintained at 37 °C for two experimental protocols: cell cultures were washed with control media and treated for (1) 2–5 min with EGTA (1 mM, a calcium chelator), control or high K^+^ media, or (2) treated with 2–3 min with control media, 50–60 μM NMDA, or APV/NMDA (50 μM APV, an NMDA receptor antagonist, in NMDA media), and then fixed immediately with 4% paraformaldehyde (EMS, Fort Washington, PA) in PBS for 20–45 min at room temperature and then washed in PBS and stored at 4 °C.

### Antibodies

Mouse monoclonal antibody against α-CaMKII (clone 6G9(2), used at 1:100) was from Millipore (Billerica, MA). Mouse monoclonal antibody against pan Shank (clone N23B/49, which recognizes all three members of the Shank family: Shank 1, 2 and 3, used at 1:250), and Shank 2 (clone N23B/6, used at 1:200) were from NeuroMab (Davis, CA). Rabbit polyclonal antibody against Shank 3 (used at 1:200–800) was from Synaptic Systems (Goettingen, Germany). Rabbit polyclonal antibody against NR2A/B (against C-terminus, at 1:100) was from Chemicon (Temecula, CA).

### Pre-embedding immunogold labelling and electron microscopy

All steps were carried out at room temperature unless otherwise indicated. Cells were permeabilized and blocked with 0.1% saponin and 5% normal goat serum in PBS for 30–60 min, incubated with primary antibodies for 1 h, washed and incubated with secondary antibodies conjugated with a small gold particle (1.4 nm-sized Nanogold from Nanoprobes, Yaphand, NY) for 1 h, washed and fixed with 2% glutaraldehyde in PBS, stored in fixative at 4 °C until ready for silver enhancement. Samples were washed in deionized water, then silver enhanced (HQ kit from Nanoprobes) at room temperature to let silver build around the small gold particles. It should be noted that the size of silver-enhanced particles is highly heterogeneous, and that samples processed on different dates may yield further differences in particle size due to the temperature-sensitive nature of the silver enhancement process.

Silver-enhanced samples were then treated with 0.2% osmium tetroxide in 0.1 M phosphate buffer at pH 7.4 on ice for 30 min, followed by treatment with 0.25% uranyl acetate in 0.1 N acetate buffer at pH 5.0 on ice for 30–60 min, then dehydrated through a series of ethanol and finally embedded in epoxy resins. Thin sections of 70–90 nm were cut *en face* and counter-stained with uranyl acetate and lead citrate. Images were taken with a digital CCD camera (AMT XR-100, Danvers, MA, USA).

### Morphometry

#### Scoring of different types of distribution pattern of label for CaMKII

At least 4–5 grid openings (400-mesh honeycomb-patterned grids) were randomly chosen from each thin-sectioned sample and every cross-sectioned asymmetric synapse encountered were photographed and scored based on the distribution patterns of label for CaMKII at the PSD. It should be noted that synapses sampled in dissociated cultures are derived from a mixture of neurons from different regions of the hippocampus, where different types of glutamatergic synapses exist. These synapses are of different sizes and shape [14], but all displayed similar stimulation-induced translocation of CaMKII to the PSD (personal observation based on materials from ref. # 6).

**Fig. 1:**
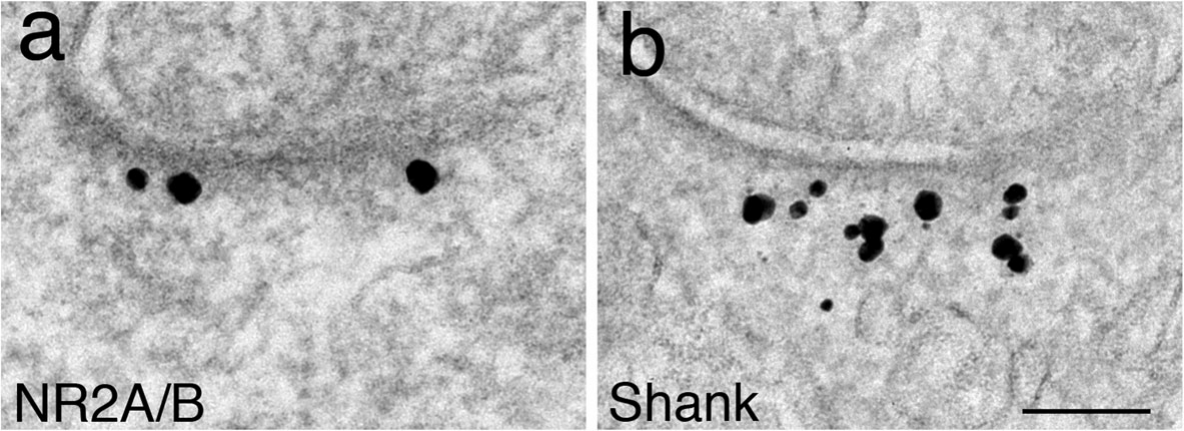
The two layers of the PSD are specifically labeled with different antibodies. For example, label for NR2A/B is in the PSD core (**a**), and label for Shank is mostly in the PSD pallium (**b**) [8]. Dark particles of heterogenous sizes are silver enhanced gold particles representing signals of the immunolabeling. Both samples were under control conditions. Scale bar = 100 nm

The PSD contains two layers: (1) the PSD core, the ~ 30 nm thick of dark material underneath the postsynaptic membrane, and (2) the PSD pallium, which is the deeper layer of the PSD, further extending from the edge of the PSD core into the cytoplasm [7]. Figure 1 illustrates the two different layers by immunolabeling of two specific antibodies localized to the two layers, respectively.

Based on the distribution of CaMKII in PSD core, PSD pallium, and the nearby cytoplasm, the pattern of labeling was classified into five types (Types I-V) in the present study. Examples of each type and the criteria for scoring are described in detail in the first section of Results. The percentage of each type was calculated for each sample.

#### Measurement of density and distance of label for CaMKII in the PSD

**Fig. 2:**
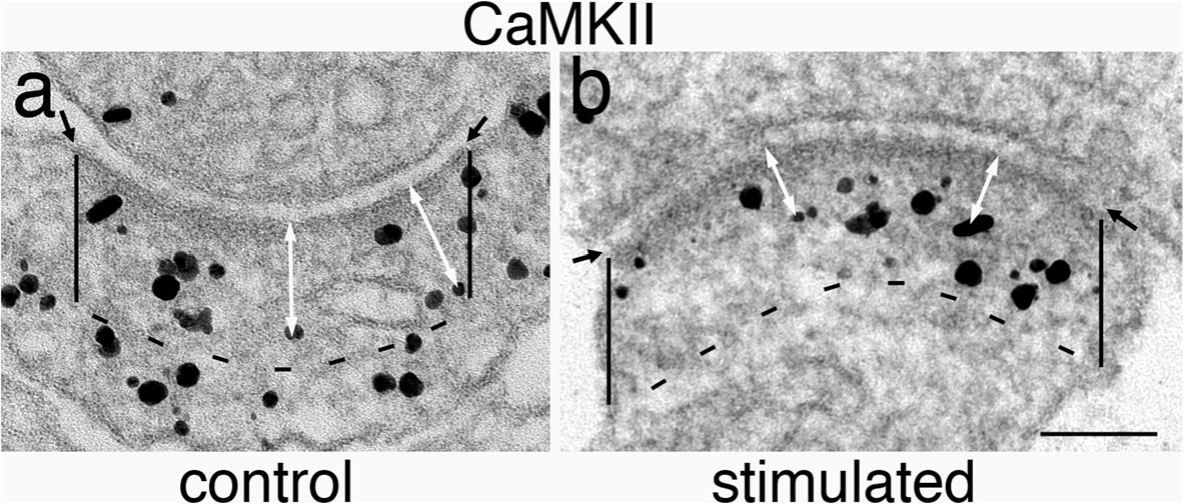
Marking PSD region for measurements. The PSD region is first outlined by the postsynaptic membrane, the edges of which were marked by two small black arrows in **a** and **b**. Two vertical lines were then drawn from the edges of the postsynaptic membrane 120 nm deep into the cytoplasm. A dashed black line parallel to the postsynaptic membrane was then drawn to enclose the PSD region for measurement. The distances of particles to postsynaptic membrane were marked by the white double-headed arrows. Sample on left (a) was treated with control media, and sample on right (b) was treated with 50 μM of NMDA for 2 min. Scale bar = 100 nm

The PSD region for measurement is arbitrarily marked as the cytoplasmic area extending 120 nm beneath the postsynaptic membrane (Fig. 2). One example each of synapses under control or stimulated condition is illustrated. Labeling density at the PSD is defined as number of gold particles within the 120 nm depth of the PSD (including both the core and the pallium), divided by the length of the marked area. The distance of each gold particle within the marked area of PSD was measured from the center of the gold particle to the outer edge of the postsynaptic membrane (white arrows in Fig. 2). All distance measurements from a sample were plotted into a histogram to illustrate the laminar distribution of label from the postsynaptic plasma membrane.

The cytosolic boundary of the PSD pallium is typically indistinct under control conditions (Fig. 2a) and appears irregular and generally within 120 nm to the postsynaptic membrane under excitatory conditions [15] (Fig. 2b). The depth for measurement was set at 120 nm in order not to undercount labels associated with the PSD. However, this measurement criteria could include some labels that may not be PSD-related, especially for control samples (Fig. 2a). Thus, for control samples, labeling density of PSD and some of the distal labels in histograms may be overcounted. On the other hand, in stimulated samples, most label for CaMKII are confined in the PSD pallium (Fig. 2b), and thus, the great majority of labels in the measurement area would be PSD-associated. Notably, the cytosolic border of the PSD pallium is irregular in shape and the pallium may not fill the boxed measurement area (Fig. 2b). Additionally, the thickness of pallium is also variable among different synapses [15].

### Statistical analysis

Statistical significance of the differences in mean values of labeling density between two groups were tested by Student’s t test, and comparisons among three groups were tested by one-way ANOVA with Tukey’s post-test. Because the distribution of distance of label is typically skewed, the significance of the differences in the median values between groups was assessed by Wilcoxon rank-sum test (KaleidaGraph, Synergy Software, Reading, PA).

## Results

Dissociated hippocampal neuronal cultures were labeled for CaMKII under different experimental conditions and examined by electron microscopy. Low calcium conditions include samples treated with calcium-free media (EGTA-treated samples), or with an NMDA receptor antagonist that blocks calcium entry (APV/NMDA-treated samples). Stimulated conditions include depolarization with high K^+^ media or treatment with NMDA. Both treatments will induce an intracellular calcium rise which will affect the distribution of CaMKII. Although these stimulation conditions may not be physiological, there are no structural damage to the cells and the effects on translocation of CaMKII are reversible [4, 5].

### Five types of CaMKII distribution patterns in the postsynaptic compartment

**Fig. 3:**
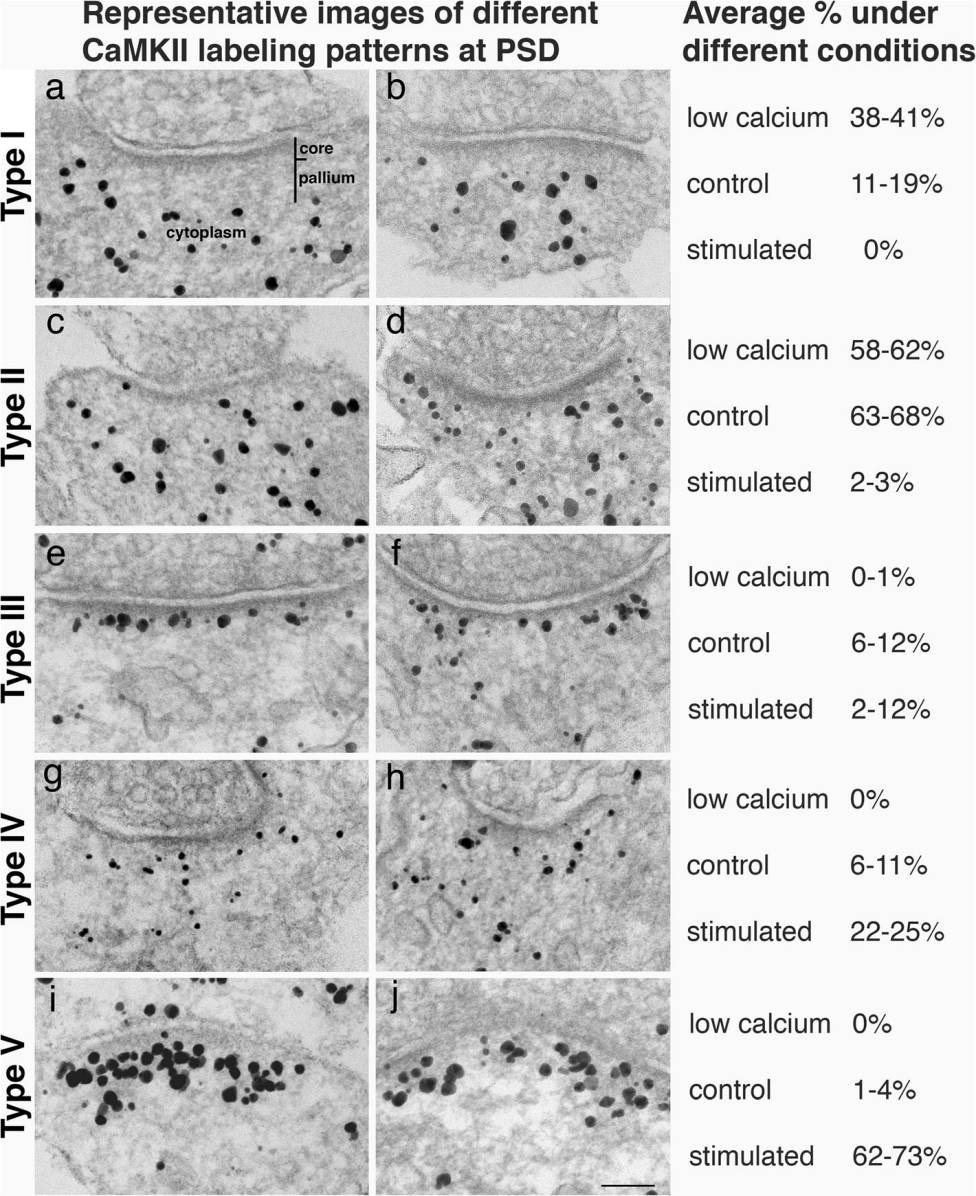
Representative images of five types of CaMKII distribution pattern at the PSD. Electron micrographs of dissociated hippocampal cultures immunogold labeled with an antibody against α-CaMKII. The percentages of each type present in different experimental conditions are listed in the right column (data from Additional Files 1 and 2). In Type I (**a**, **b**), label was absent in both the PSD core and pallium, and only present in the nearby cytoplasm. In Type II (**c**, **d**), Label was evenly distributed in the PSD and cytoplasm. In Type III (**e**, **f**), label was preferentially localized in the PSD core. In Type IV (**g**, **h**), Label was preferentially in the PSD pallium. In Type V (**i**, **j**), label was concentrated at the PSD, confined within the distal border of the pallium. Samples were under low calcium conditions (**a** and **c**, EGTA), control conditions (**b**, **d**, **e**, **f**, **g**, **h**, Ringer treatment), and stimulated conditions (**i**, high K^+^; and **j**, NMDA). Scale bar = 100 nm

Regardless of the experimental conditions, the distribution of CaMKII for synapses sampled was classified into five types based on the presence of label for CaMKII in PSD core, PSD pallium and the nearby cytoplasm:

- ***Type I. “Not at the PSD”*** (Fig. 3a, b) – Label is absent in the PSD (including both the core and the pallium), and only present in the cytoplasm surrounding the PSD. Although there is no detectible structural distinction between the PSD pallium and the cytoplasm, the interface between the PSD pallium and the cytoplasm is delineated by the lack of gold particles in the PSD. This labeling pattern of CaMKII avoiding the PSD also suggest that the composition in the PSD pallium is different from that of the neighboring cytoplasm for CaMKII binding.
- ***Type II. “Evenly in the PSD and cytoplasm”*** (Fig. 3c, d) – Label is evenly dispersed in the PSD as well as in the nearby cytoplasm. There is no conspicuous border between the distal edge of the PSD pallium and the cytoplasm.
- ***Type III. “Lined up near PSD core”*** (Fig. 3e, f) – Label is preferentially localized near the border of PSD core at ~ 30 nm from the postsynaptic membrane. There may be other gold particles in the PSD pallium or in the cytoplasm, but there is no apparent structural border between the distal edge of the PSD pallium and the cytoplasm.
- ***Type IV. “In the pallium”*** (Fig. 3g, h) – Label is preferentially in the PSD pallium, with some labels in the cytoplasm. There is no clear-cut structural distinction between the distal edge of the PSD pallium and the cytoplasm, but the border is to some extent delineated by the gold particles in the pallium.
- ***Type V. “Concentrated at the PSD”*** (Fig. 3i, j)– Label is concentrated in the PSD, particularly at the pallium, with very little labeling in the cytoplasm. The border between PSD pallium and cytoplasm is sharply defined by the edge of the concentrated gold particles.

### The different types of postsynaptic CaMKII distribution are related to the activity states of the synapses

**Fig. 4:**
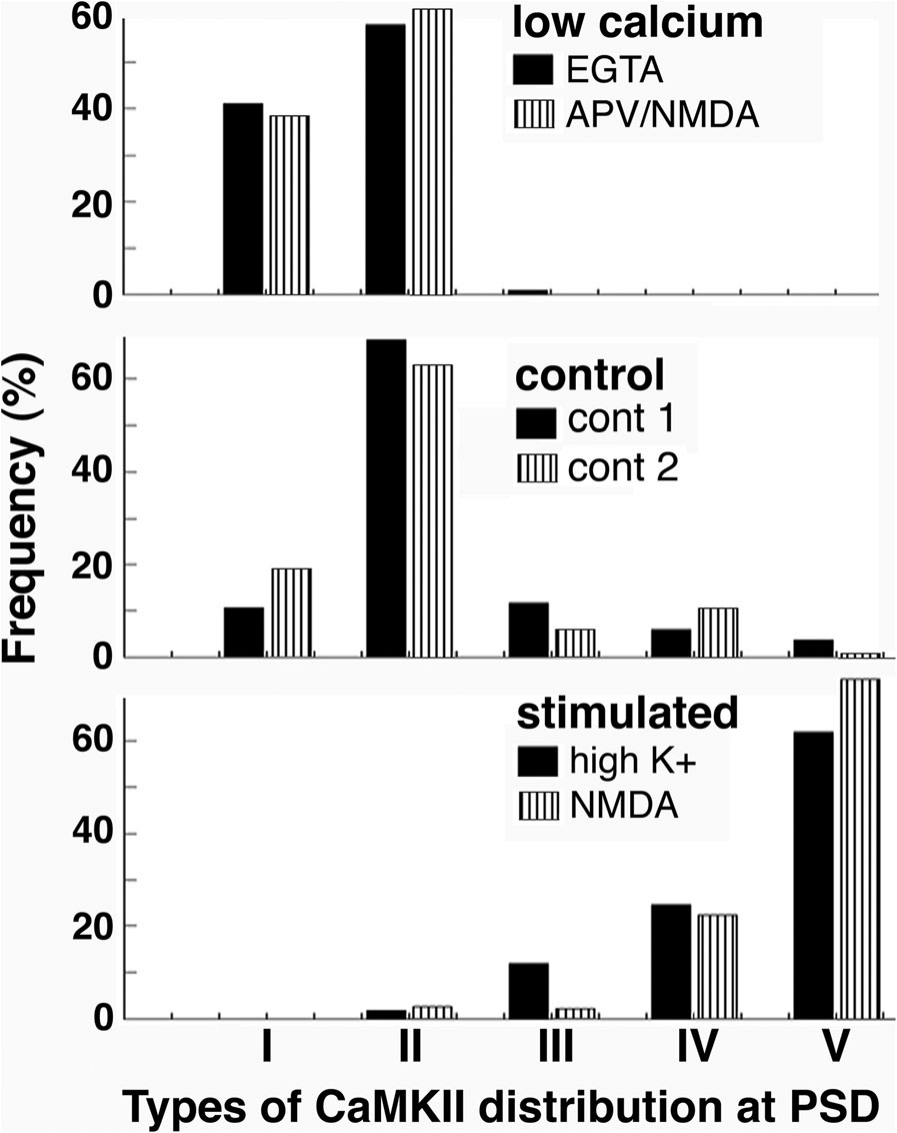
Occurrence frequencies of the five types of CaMKII distribution at the PSD under different experimental conditions. Types I and II are predominantly present in samples under low calcium and control conditions, while Type V is predominantly present in stimulated samples. Type II and III may represent intermediate stages of CaMKII distribution between control and stimulated conditions. Bars represent mean values from Additional Files 1 and 2

Every cross-sectioned, CaMKII-labeled synaptic profile encountered was classified into one of the above mentioned five types of labeling patterns. The percentages of each type were tabulated for each sample within each experiment (Additional Files 1 & 2), and mean values for each type were calculated for each experimental conditions. Bar graphs in Fig. 4 shows the mean values of each type under the three experimental conditions (low calcium, control, stimulated).

The mean occurrence frequency of Type I (not at the PSD) of CaMKII distribution was higher (38–41%, top panel of Fig. 4, first column on left) in low calcium samples than in control samples (11–19%, middle panel of Fig. 4) and absent in stimulated samples (bottom panel of Fig. 4). Since stimulation leads to calcium entry and a rise in intracellular calcium concentration [1, 2], the frequency of Type I appears to be in reverse relationship to calcium concentration.

The Type II (evenly in PSD and cytoplasm) distribution pattern of CaMKII is frequently seen in samples under low calcium (58–62%) or control conditions (63–68%) and rarely seen in stimulated samples (2–3%). In reverse proportion, Type V (concentrated at the PSD) was absent in low calcium samples, rarely seen (1–4%) in control samples, and predominant (62–73%) in stimulated samples. Thus, Type II is prominent when calcium is at low and control levels, while Type V is linked with high calcium levels induced by stimulation.

Type III (lined up near PSD core) and IV (in PSD pallium) were relatively low in overall frequency compare to other types. They were rarely seen in low calcium samples, and appeared at low average frequencies in control, and low to medium frequencies in stimulated samples. It is possible that these two patterns of CaMKII distribution represent synapses in intermediate stages between basal and stimulated conditions, and that these patterns may transiently appear at intermediate levels of calcium concentrations.

### Laminar distribution of label for CaMKII at the PSD changes with activity

**Fig. 5:**
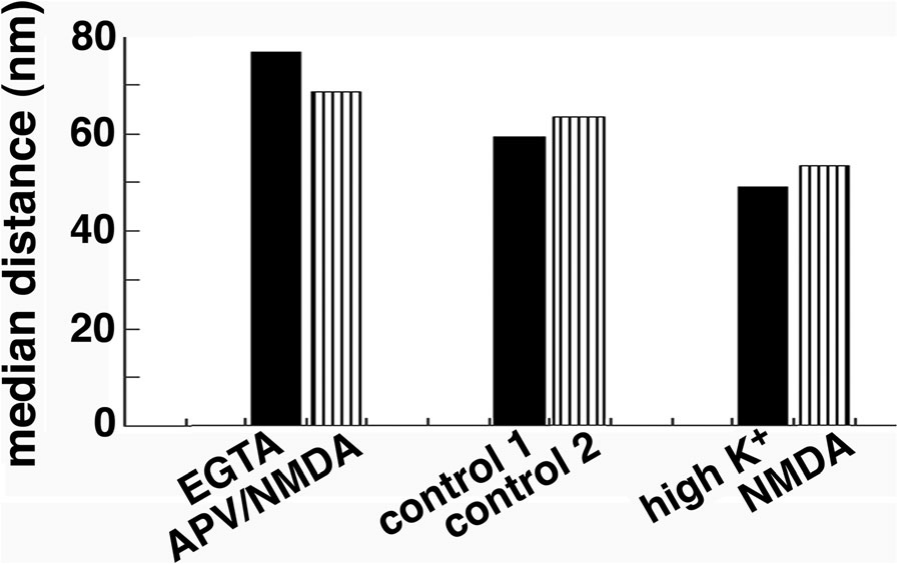
Median distance of label for CaMKII from postsynaptic membrane (nm) under different experimental conditions. Bars represent mean values from Additional Files 3 and 4

In order to quantify the activity-related redistribution of CaMKII, the distances of label from the postsynaptic membrane were measured (Additional files 3 & 4). Median distance of label for CaMKII to the postsynaptic membrane was higher in low calcium samples (68–77 nm), than in control (59–63 nm) and stimulated samples (49–53 nm; Fig. 5). These results indicate that CaMKII molecules were overall located further away from the postsynaptic membrane under low calcium conditions, but closer to the postsynaptic membrane upon stimulation.

**Fig. 6:**
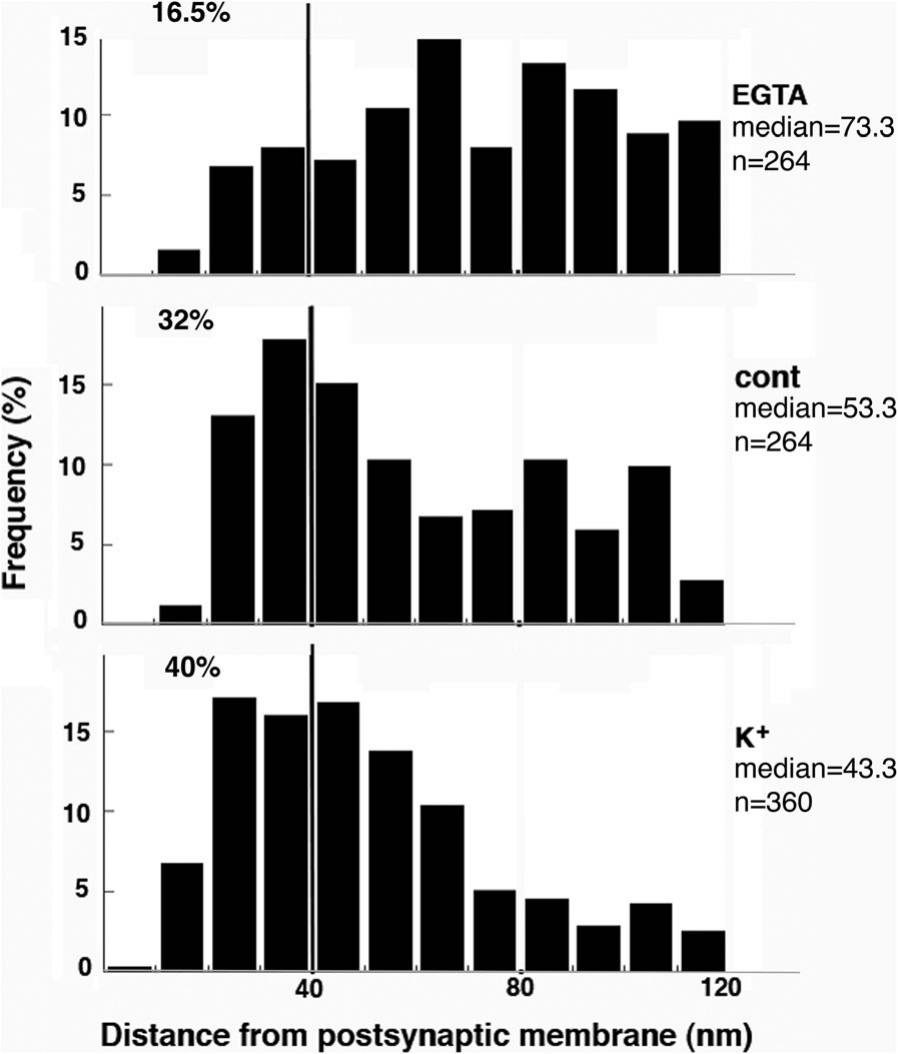
Histograms of distance of label for CaMKII from the postsynaptic membrane (data from experiment 1 in Additional File 3). Median distance progressively decreased from low calcium conditions (EGTA, top panel) to control (middle panel) and stimulated conditions (K^+^, bottom panel). The vertical line at 40 nm divides the PSD core (left) from the PSD pallium (right), and percentages of label in PSD core progressively increased from top to bottom

Histograms of distance data from one representative experiment further showed fewer CaMKII label within 40 nm of the postsynaptic membrane under EGTA treatment (16.5%, top panel of Fig. 6) than under control conditions (32%, middle panel of Fig. 6). This differences in laminar distribution of CaMKII between control and low calcium samples are statistically significant in all three experiments (Additional File 3), indicating that some CaMKII molecules move out of the PSD core under low calcium conditions. However, there was still a persistent amount (about half) of CaMKII molecules that remained in the PSD core under low calcium conditions when compared to controls. On the other hand, more CaMKII labels were within 40 nm of the postsynaptic membrane under high K^+^ treatment (40%; bottom panel of Fig. 6) than controls. This difference reached statistical significance in two out of three experiment (Additional file 3). Considering that labeling density of CaMKII was significantly higher in high K^+^ samples (Additional File 5), these results strongly suggest that more CaMKII molecules are recruited into the PSD core upon stimulation.

Similarly, upon NMDA treatment, labeling density of CaMKII at the PSD significantly increased (Additional File 6), with a decrease in median distance of label for CaMKII (Additional File 4) from postsynaptic membrane. Histograms of distance measurements further indicate that there were more CaMKII within 40 nm of the postsynaptic membrane in NMDA-treated samples than controls (Additional File 7).

### The distribution of label for CaMKII and shank at the PSD were different under control conditions, but became similar upon stimulation

**Fig. 7:**
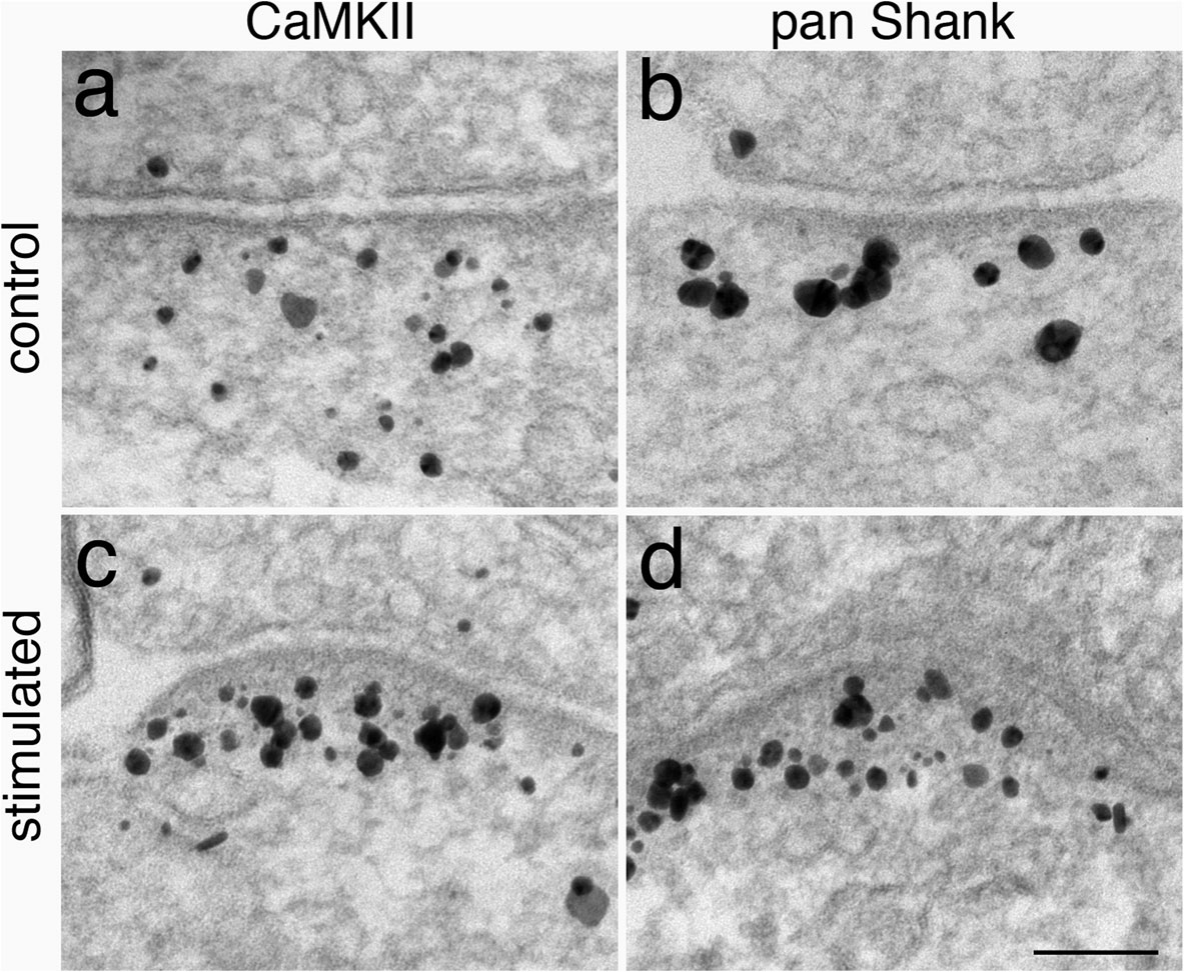
Distribution of CaMKII (**a**, **b**) and Shank (using a pan Shank antibody, **c**, **d**) under control (**a**, **c**) and NMDA treatment (**b**, **d**)

Because PSD pallium (the deeper layer of PSD) shows the most dynamic changes upon stimulation [7], the distribution of label for CaMKII was compared to that for Shank, a prominent scaffold protein in the PSD pallium [11–13], under different experimental conditions to examine possible interactions between these molecules. Under control conditions where synapses were mostly under basal state, CaMKII was typically distributed evenly in the PSD as well as in the nearby cytoplasm (Fig. 7a), while Shank was mostly localized in the PSD pallium (Fig. 7b). However, upon stimulation, both CaMKII (Fig. 7c) and Shank (Fig. 7d) became colocalized in the PSD, with translocation of additional molecules from the nearby cytoplasm to the PSD, mostly at the PSD pallium [4, 12]. This pattern of activity-dependent redistribution of CaMKII and Shank was also observed when antibodies specific for subtypes of Shank 2 and Shank 3 were used (Additional File. 8). Most importantly, upon stimulation, labels for CaMKII and Shank essentially did not extend beyond the distal border of the pallium (Fig. 7c, d; Additional File 8 g, h, i).

To further compare their laminar distribution, distance measurement of label for CaMKII and Shank were carried out from sister cultures treated with high K^+^ media for 2 min. Histograms showed similarly skewed distribution with a wide range from 7 to 120 nm, and those from one representative experiment are shown in Additional File 9. There were no significant differences in median distance between the labels for CaMKII and Shank 2, and a slight difference in 2 out of 3 experiments between the labels for CaMKII and pan Shank (Additional File 10). These results illustrate that the bulk of labels for CaMKII and Shank are colocalized in the PSD pallium upon stimulation.

## Discussion

The present study examined the distribution patterns of CaMKII in dissociated hippocampal cultures under different conditions, with particular interests on their laminar distribution within the postsynaptic compartment and their potential association with other PSD proteins that are localized to different layers of the PSD.

CaMKII is known to be dispersed in the postsynaptic compartment under basal conditions and translocate to the PSD upon stimulation [4–6]. Here, distance measurements of label for CaMKII from the postsynaptic membrane indicate that the laminar distributions of CaMKII are significantly different under the three experimental conditions: compared to controls, the median distances increased under low calcium conditions (EGTA or APV/NMDA), and decreased upon stimulation (K^+^ or NMDA treatment). Thus, some CaMKII molecules moved away from the postsynaptic membrane under low calcium conditions, and many CaMKII molecules moved closer to the postsynaptic membrane upon stimulation, implying preferential binding to different proteins under different conditions.

Is this activity-dependent redistribution of CaMKII into different layers of the PSD only present in dissociated cultures and not in brains? The presence of pallium, the deeper layer of PSD, was demonstrated in brain tissues with the labeling of Shank and CaMKII at distances of up to 100 nm to the postsynaptic membrane [8, 9]. Furthermore, two previous studies on perfusion-fixed brains showed translocation of CaMKII to the PSD upon ischemia-like excitation due to a few minutes delay in perfusion fixation [6, 10]. Thus, glutamatergic synapses in vitro and in vivo exhibited similar pattern of stimulation-induced translocation of CaMKII to the PSD.

Among the known binding partners for CaMKII in the postsynaptic compartment, NR2B [8], densin [16] and α-actinin [17] are localized in the PSD core (defined here as within 40 nm to the postsynaptic membrane), Shank [8, 9, 12] and CYLD [18] are mostly localized in the PSD pallium (40–120 nm to the postsynaptic membrane), and F-actin [19, 20] is in the cytoplasm (beyond 120 nm from the postsynaptic membrane). Under low calcium conditions, CaMKII may be held in the cytoplasm by binding to the actin cytoskeleton [1], resulting in an increase in frequency of Type I CaMKII distribution pattern where CaMKII avoids the PSD in ~ 40% of synapses examined. The CaMKII molecules that move out of the PSD under low calcium conditions likely results from uncoupling of CaMKII from NR2B because their binding is calcium-dependent [1, 2]. Similarly, upon treatment of a cell-permeable CaMKII inhibitor, tatCN21, which disrupts the binding between NR2B and CaMKII [1], the type I distribution of CaMKII was also more frequently observed than in control samples [21]. However, it should be noted that even under low calcium conditions, there is still overall a low but persistent presence of CaMKII at the PSD core, perhaps from CaMKII molecules binding to densin or α-actinin [1, 2, 17].

In control samples, the great majority of synapses exhibited Type II pattern of CaMKII distribution with labels dispersed in the postsynaptic compartment, where CaMKII may be evenly associated with binding partners throughout the spine including the PSD and the cytoplasm. Upon stimulation with an accompanying calcium rise, CaMKII molecules are released from their binding with actin cytoskeleton in the cytoplasm and translocate to the PSD [1, 2]. The translocated CaMKII molecules could initially bind to NR2B [22], densin [23] or α-actinin [24] in the PSD core, appearing as a line of preferential labeling near the distal edge of the PSD core (Type III of CaMKII distribution presented here).

It is also possible that under certain excitatory conditions, the translocated CaMKII molecules initially bind to some proteins in the PSD pallium, resulting as Type IV of CaMKII distribution presented here. The most likely candidate for such a CaMKII binding partner in PSD pallium is Shank 3, a major PSD scaffold protein mostly located in the PSD pallium [13], which is abundantly associated with CaMKII [25], and which co-immunoprecipitates with activated CaMKII [26], a finding demonstrating a novel, activity-dependent binding of CaMKII and Shank 3 [26]. A second, much weaker candidate is CYLD, a deubiquitinase localized at the PSD, which also co-immunuprecipatates with CaMKII [18]. However, the amount of CYLD at the PSD is only ~ 8% of that of Shank 3 [27], making CYLD a minor player in CaMKII binding at the PSD compared to Shank 3. On the other hand, there is no evidence of Homer and IRSp53, two other PSD proteins that are located in the PSD pallium [28, 29], interacting directly with CaMKII.

It should be noted that Type III & IV of CaMKII distribution (the two proposed intermediate patterns) were low frequency events, perhaps due to their transitory nature between basal and excited states. The fact that neither type was present in samples of low calcium conditions indicates that both types require an above-threshold calcium rise, which is most likely induced by synaptic activity. It is not clear whether Type III and IV distribution patterns are independent or sequential events, or whether the different responses are due to different levels of calcium rise or due to different types of synapses from mixed neuronal types in dissociated hippocampal cultures. Nevertheless, both types may represent the initial stages of activity-induces translocation of CaMKII to the PSD.

Notably, the great majority of synapses in highly stimulated samples were of Type V, where label for CaMKII were greatly concentrated at the PSD (including both the core and the pallium). Although NR2B at the PSD core has been identified as an important player in stimulus-induced translocation of CaMKII to the PSD, the vast amount of translocated CaMKII suggests that other PSD proteins also take part in recruiting CaMKII to the PSD [2]. Furthermore, label for antibodies made against the C-terminal of NR2B, a binding site to CaMKII, is localized within the PSD core [8]. Thus, CaMKII molecules in the distal region of the PSD pallium are too far away to be directly binding to NR2B. The additional CaMKII molecules recruited to the PSD pallium upon heightened stimulation could be binding to other CaMKII molecules via CaMKII self-clustering [30, 31]. However, it should be noted that label for CaMKII did not extend beyond the border of pallium, suggesting a limiting mechanism that prevents CaMKII to self-cluster beyond the pallium. On the other hand, in view of the abundant binding of CaMKII and Shank 3 [25, 26], upon stimulation, the translocated CaMKII may be binding to resident and recruited Shank 3 molecules at the PSD pallium. This speculation awaits verification of future experiments with Shank3-knockout mice or with an in vitro cell culture system. Nevertheless, as both Shank and CaMKII co-localize at the PSD with very similar patterns upon stimulation, it is possible that Shank is the binding partner that confines CaMKII in the pallium under excitatory conditions.

## Supplementary information

**Additional file 1.**

**Additional file 2.**

**Additional file 3.**

**Additional file 4.**

**Additional file 5.**

**Additional file 6.**

**Additional file 7.**

**Additional file 8.**

**Additional file 9.**

**Additional file 10.**

## Data Availability

The datasets generated and/or analyzed during the current study are available from the corresponding author on reasonable request.
