# Supplementary material for: Activity-dependent redistribution of CaMKII in the postsynaptic compartment of hippocampal neurons
Source: Mol Brain. 2020 Apr 1;13:53. doi: 10.1186/s13041-020-00594-5 (PMC7110642; doi:10.1186/s13041-020-00594-5)
Supplement: Supplementary file 1 — Additional file 1. [file 13041_2020_594_MOESM1_ESM.docx]

**Additional File 1. Percent frequency (%) of each type of CaMKII distribution**

**at the PSD upon low calcium and depolarizing conditions.**

|  | | **Type I.** Not at the PSD | **Type II.** Evenly in PSD and cytoplasm | **Type III.** Lined up near PSD core | **Type IV.**  In PSD pallium | **Type V.** Concentrated at PSD | Total # PSD scored |
| --- | --- | --- | --- | --- | --- | --- | --- |
| **EGTA** | Exp 1 | 37.2 | 58.1 | 4.7 | 0 | 0 | 43 |
|  | Exp 2 | 50 | 50 | 0 | 0 | 0 | 24 |
|  | Exp 3 | 41.7 | 58.3 | 0 | 0 | 0 | 36 |
|  | Exp 4 | 40 | 60 | 0 | 0 | 0 | 20 |
|  | Exp 5 | 35.9 | 64.1 | 0 | 0 | 0 | 39 |
|  | **Mean±SEM** | **41.0±2.5** | **58.1±2.3** | **0.9±0.3** | **0** | **0** |  |
| **control** | Exp 1 | 12.5 | 72.5 | 15 | 0 | 0 | 40 |
|  | Exp 2 | 9.5 | 66.7 | 9.5 | 14.3 | 0 | 21 |
|  | Exp 3 | 15.2 | 58.7 | 6.5 | 8.7 | 10.9 | 46 |
|  | Exp 4 | 16.7 | 66.7 | 16.7 | 0 | 0 | 12 |
|  | Exp 5 | 3.3 | 76.7 | 6.7 | 3.3 | 10 | 30 |
|  | Exp 6 | 5.9 | 41.2 | 29.4 | 17.6 | 5.9 | 17 |
|  | Exp 7 | 22.7 | 77.3 | 0 | 0 | 0 | 22 |
|  | Exp 8 | 0 | 87.0 | 8.7 | 4.3 | 0 | 23 |
|  | **Mean±SEM** | **10.7±2.7** | **68.3±4.9** | **11.6±3.1** | **6.0±2.4** | **3.8±1.7** |  |
| **K^+^** | Exp 1 | 0 | 0 | 27.6 | 13.8 | 58.6 | 29 |
|  | Exp 2 | 0 | 7.4 | 7.4 | 33.3 | 51.9 | 27 |
|  | Exp 3 | 0 | 0 | 0 | 47.1 | 52.9 | 17 |
|  | Exp 6 | 0 | 3.6 | 0 | 0 | 96.4 | 28 |
|  | Exp 7 | 0 | 0 | 6.3 | 37.5 | 56.2 | 32 |
|  | Exp 8 | 0 | 0 | 30 | 15 | 55 | 20 |
|  | **Mean±SEM** | **0** | **1.8±1.3** | **11.9±5.5** | **24.5±7.2** | **61.8±7.0** |  |
