# Supplementary material for: Activity-dependent redistribution of CaMKII in the postsynaptic compartment of hippocampal neurons
Source: Mol Brain. 2020 Apr 1;13:53. doi: 10.1186/s13041-020-00594-5 (PMC7110642; doi:10.1186/s13041-020-00594-5)
Supplement: Supplementary file 2 — Additional file 2. [file 13041_2020_594_MOESM2_ESM.docx]

**Additional File 2. Percent frequency (%) of each type of CaMKII distribution**

**at the PSD upon NMDA treatment.**

|  | | **Type I.** Not at the PSD | **Type II.** Evenly in PSD and cytoplasm | **Type III.** Lined up near PSD core | **Type IV.**  In PSD pallium | **Type V.** Concentrated at PSD | Total # PSD scored |
| --- | --- | --- | --- | --- | --- | --- | --- |
| **control** | Exp 1 | 21.3 | 60.7 | 7.1 | 10.7 | 0 | 28 |
|  | Exp 2 | 22.2 | 55.6 | 5.5 | 11.1 | 5.5 | 18 |
|  | Exp 3 | 41.7 | 50 | 0 | 8.3 | 0 | 12 |
|  | Exp 4 | 0 | 71.4 | 14.3 | 14.3 | 0 | 21 |
|  | Exp 5 | 10.3 | 77.9 | 2.9 | 8.8 | 0 | 68 |
|  | **Mean±SEM** | **19.1±7.0** | **63.1±5.1** | **6.0±2.4** | **10.6±1.1** | **1.1±1.1** |  |
| **NMDA** | Exp 1 | 0 | 0 | 0 | 21.1 | 78.9 | 38 |
|  | Exp 2 | 0 | 12.8 | 10.6 | 21.3 | 55.3 | 47 |
|  | Exp 3 | 0 | 0 | 0 | 33.3 | 66.7 | 12 |
|  | Exp 4 | 0 | 0 | 0 | 5.6 | 94.4 | 36 |
|  | Exp 5 | 0 | 0 | 0 | 30.4 | 69.6 | 56 |
|  | **Mean±SEM** | **0** | **2.6±2.6** | **2.1±2.1** | **22.3±4.8** | **73.0±6.5** |  |
| **APV/**  **NMDA** | Exp 1 | 39.3 | 60.7 | 0 | 0 | 0 | 28 |
|  | Exp 2 | 37.5 | 62.5 | 0 | 0 | 0 | 24 |
|  | **Mean±SEM** | **38.4±0.9** | **61.6±0.9** | **0** | **0** | **0** |  |
