# Supplementary material for: Activity-dependent redistribution of CaMKII in the postsynaptic compartment of hippocampal neurons
Source: Mol Brain. 2020 Apr 1;13:53. doi: 10.1186/s13041-020-00594-5 (PMC7110642; doi:10.1186/s13041-020-00594-5)
Supplement: Supplementary file 3 — Additional file 3. [file 13041_2020_594_MOESM3_ESM.docx]

**Additional File 3. Mean and median distances (nm) of CaMKII labels from postsynaptic membrane upon low calcium and depolarizing conditions.**

|  | **EGTA** | | **control** | | **High K^+^** | |
| --- | --- | --- | --- | --- | --- | --- |
|  | **mean** | **median** | **mean** | **median** | **mean** | **median** |
| **Exp 1** | 72.8±1.8  (264) | 73.3 | 60.6±1.8  (264) | 53.3 | 49.2±1.4  (360) | 43.3 |
| **Exp 2** | 80.1±2.4  (136) | 83.3 | 60.5±2.5  (147) | 56.7 | 57.3±1.7  (243) | 53.3 |
| **Exp 3** | 75.2±2.4  (137) | 73.3 | 58.2±2.1  (189) | 53.3 |  |  |
| **Exp 7** |  |  | 72.3±2.9  (98) | 73.3 | 53.2±1.7  (202) | 50 |
| **Mean±SEM** |  | **76.6±3.3** |  | **59.2±4.8** |  | **48.9±2.9** |

Experiment numbers are the same as in Additional File 1.

(n=number of particles measured)

Statistical analyses by Wilcoxon test for medians within each experiment:

Exp 1: P<0.0001, EGTA vs. control; control vs. high K^+^; EGTA vs. high K^+^.

Exp 2: P<0.0001, EGTA vs. control; EGTA vs. high K^+^.

Exp 3: P<0.0001, EGTA vs. control.

Exp 7: P<0.0001, control vs. high K^+^.
