# Supplementary material for: Activity-dependent redistribution of CaMKII in the postsynaptic compartment of hippocampal neurons
Source: Mol Brain. 2020 Apr 1;13:53. doi: 10.1186/s13041-020-00594-5 (PMC7110642; doi:10.1186/s13041-020-00594-5)
Supplement: Supplementary file 4 — Additional file 4. [file 13041_2020_594_MOESM4_ESM.docx]

**Additional File 4. Mean and median distances (nm) of CaMKII labels from postsynaptic membrane upon NMDA treatment.**

|  | **control** | | **NMDA** | | **APV/NMDA** | |
| --- | --- | --- | --- | --- | --- | --- |
|  | **mean** | **median** | **mean** | **median** | **mean** | **median** |
| **Exp 1** | 69.3±2.7  (120) | 66.7 | 59.9±1.5  (358) | 56.7 | 69.3±2.8  (109) | 66.7 |
| **Exp 2** | 61.4±2.2  (157) | 60 | 57.4±1.3  (446) | 53.3 | 73.1±2.8  (82) | 70 |
| **Exp 5** | 65.2±1.9  (225) | 63.3 | 51.7±1.7  (217) | 46.7 |  |  |
| **Mean±SEM** |  | **63.3±1.9** |  | **53.3±2.9** |  | **68.3±1.7** |

Experiment numbers are the same as in Additional File 2.

(n=number of particles measured)

Statistical analyses by Wilcoxon test for medians within each experiment:

Exp 1: P<0.005, control vs. NMDA; NMDA vs. APV/NMDA.

Exp 2: P<0.1, control vs. NMDA; P<0.0001, NMDA vs. APV/NMDA; P<0.005, control vs. APV/NMDA.

Exp 5: P<0.0001, control vs. NMDA.
