# Supplementary material for: Activity-dependent redistribution of CaMKII in the postsynaptic compartment of hippocampal neurons
Source: Mol Brain. 2020 Apr 1;13:53. doi: 10.1186/s13041-020-00594-5 (PMC7110642; doi:10.1186/s13041-020-00594-5)
Supplement: Supplementary file 5 — Additional file 5. [file 13041_2020_594_MOESM5_ESM.docx]

**Additional File 5. Density of label for CaMKII at PSD under**

**Low calcium and depolarizing conditions.**

|  | **EGTA** | **control** | **High K^+^** |
| --- | --- | --- | --- |
| **Exp 1** | 34.0±3.0 [104%]  (30) | 32.6±3.3  (32) | 62.6±2.8 [192%]  (26) |
| **Exp 2** | 24.4±2.0 [94%]  (20) | 26.0±2.5  (21) | 54.0±3.9 [208%]  （23） |
| **Exp 3** | 23.2±2.5 [83%]  (21) | 28.0±2.8  (24) |  |
| **Exp 7** |  | 18.9±2.8  (14) | 31.5±2.2 [167%]  (30) |
| **Mean**  **[% control]** | **[93.5±10.5%]** | **100%** | **[179.5±12.5%]** |

Experiment numbers are the same as in Additional File 1.

Unit for density of label is number of particles/µm PSD length.

Values are listed and then normalized to [% of control values].

(n=synaptic profiles)

Statistical analyses within each experiment by ANOVA with Tukey’s post-test:

Exp 1: P<0.0001, EGTA vs. high K^+^; control vs. high K^+^.

Exp 2: P<0.0001, EGTA vs. high K^+^; control vs. high K^+^.

Statistical analyses within each experiment by Student’s test:

Exp 3: not significant, EGTA vs. control.

Exp 7: P<0.005, control vs. high K^+^.
