# Supplementary material for: Activity-dependent redistribution of CaMKII in the postsynaptic compartment of hippocampal neurons
Source: Mol Brain. 2020 Apr 1;13:53. doi: 10.1186/s13041-020-00594-5 (PMC7110642; doi:10.1186/s13041-020-00594-5)
Supplement: Supplementary file 6 — Additional file 6. [file 13041_2020_594_MOESM6_ESM.docx]

**Additional File 6. Density of label for CaMKII at PSD**

**upon NMDA treatment.**

|  | **control** | **NMDA** | **APV/NMDA** |
| --- | --- | --- | --- |
| **Exp 1** | 21.4±1.9  (17) | 45.4±4.1 [212%]  (23) | 16.5±1.9 [77%]  (19) |
| **Exp 2** | 34.0±4.5  (17) | 42.3±2.2 [124%]  (39) | 18.4±2.3 [54%]  (17) |
| **Exp 5** | 42.3±3.1  (31) | 71.1±4.6 [168%]  (36) |  |
| **Mean**  **[% control]** | **100%** | **[168±25.4%]** | **[65.5±11.5%]** |

Experiment numbers are the same as in Additional File 2.

Unit for density of label is number of particles/µm PSD length.

Values are listed and then normalized to [% of control values].

(n=synaptic profiles)

Statistical analyses within each experiment by ANOVA with Tukey’s post-test:

Exp 1: P<0.0001, control vs. NMDA; NMDA vs. APV/NMDA.

Exp 2: P=0.12, control vs. NMDA; P<0.0001, NMDA vs. APV/NMDA; P<0.01, control vs. APV/NMDA.

Statistical analyses by Student’s test:

Exp 5: P<0.0001, control vs. NMDA.
