# Supplementary material for: Activity-dependent redistribution of CaMKII in the postsynaptic compartment of hippocampal neurons
Source: Mol Brain. 2020 Apr 1;13:53. doi: 10.1186/s13041-020-00594-5 (PMC7110642; doi:10.1186/s13041-020-00594-5)
Supplement: Supplementary file 7 — Additional file 7. [file 13041_2020_594_MOESM7_ESM.pdf]

Additional File 7. Histograms of distance of label for CaMKII from the postsynaptic membrane

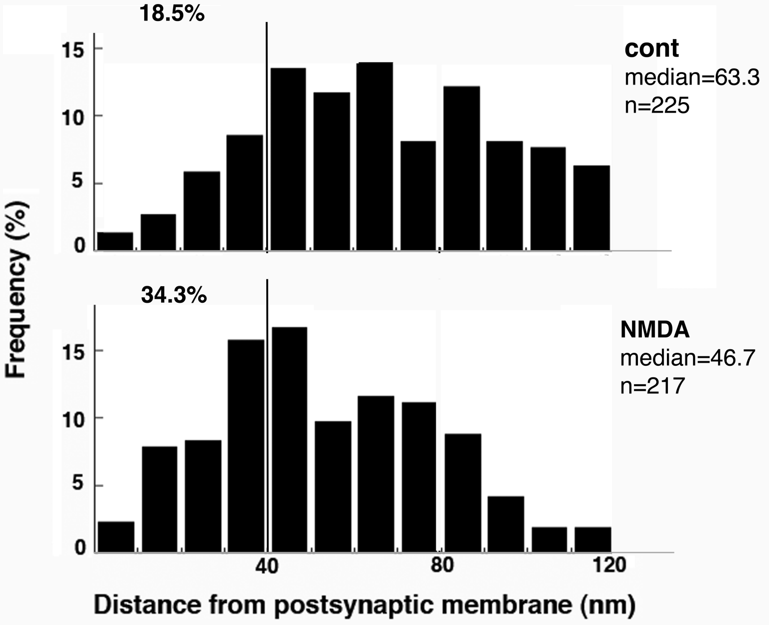

Upon NMDA treatment (lower panel), median distance decreased while occurrence frequency in the PSD core (within 40 nm of the postsynaptic membrane) increased.

(data from experiment 5 in Additional File 4)
