# Supplementary material for: Activity-dependent redistribution of CaMKII in the postsynaptic compartment of hippocampal neurons
Source: Mol Brain. 2020 Apr 1;13:53. doi: 10.1186/s13041-020-00594-5 (PMC7110642; doi:10.1186/s13041-020-00594-5)
Supplement: Supplementary file 8 — Additional file 8. [file 13041_2020_594_MOESM8_ESM.pdf]

Additional File 8. Distribution of label for CaMKII , Shank 2 and Shank 3 under EGTA treatment, control conditions and stimulated conditions.

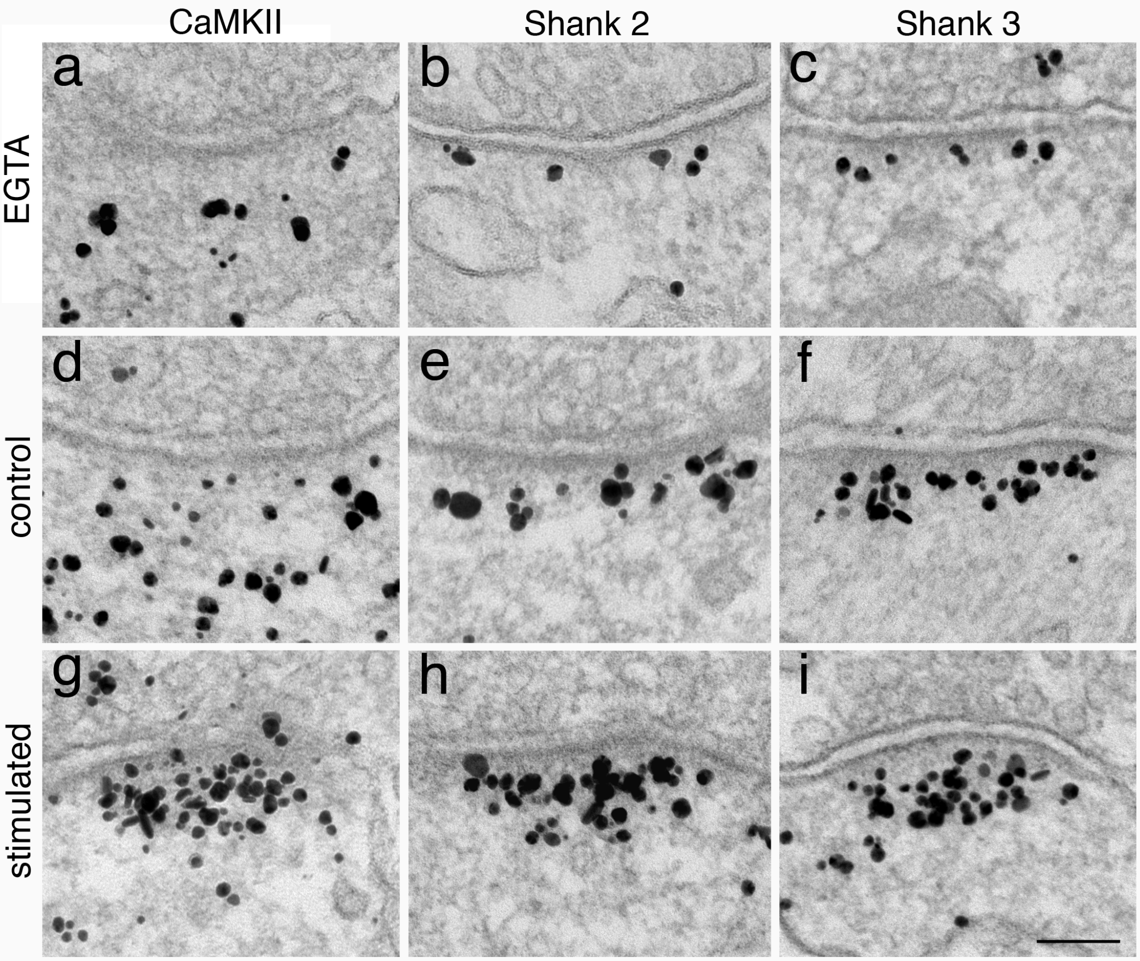

Under low calcium conditions (EGTA), ~40% of PSDs lack CaMKII (a), while the rest of synpases have labeling patterns similar to that under control conditions (d). In contrast, some PSDs contain a thin layer of Shank2 (b), or Shank3 (c) near the PSD core, while the rest of synpases have labeling patterns similar to those under control consditions (e, f). Under control conditions, the majority (>60%) of synapses had evenly distributed CaMKII in the PSD as well as in cytoplasm (d). In contrast, Shank 2 (e) and Shank 3 (f) were localized to the PSD pallium. Thus, distribution patterns for Shank 2 and Shank 3 were very similar to each other, but different from those for CaMKII under EGTA and control conditions. However, upon stimulation, label for all three antibodies became similarly aggregated at the PSD (g, h, i). Scale bar = 100 nm.
