# Supplementary material for: Activity-dependent redistribution of CaMKII in the postsynaptic compartment of hippocampal neurons
Source: Mol Brain. 2020 Apr 1;13:53. doi: 10.1186/s13041-020-00594-5 (PMC7110642; doi:10.1186/s13041-020-00594-5)
Supplement: Supplementary file 9 — Additional file 9. [file 13041_2020_594_MOESM9_ESM.pdf]

Additional File 9. Histograms of distance of label for CaMKII, pan Shank, and Shank 2 from the postsynaptic membrane.

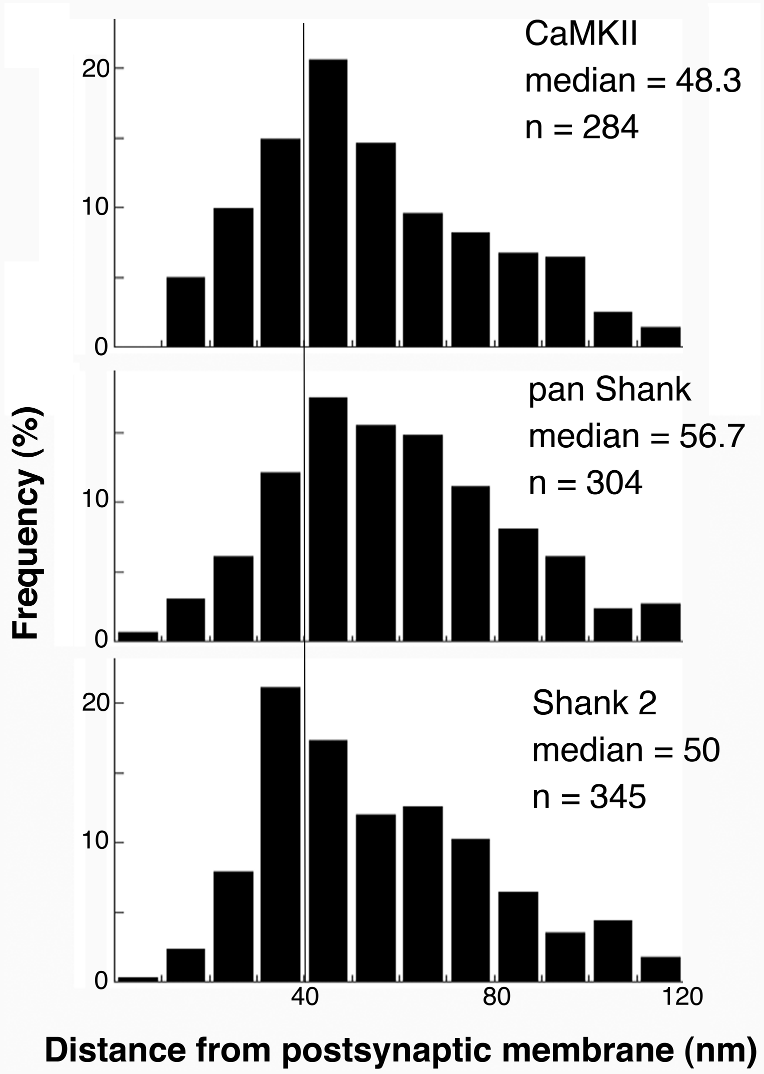

Data were from experiment 2 in Additional File 10. In this experiment, while the laminar distribution of CaMKII (top panel) and Shank 2 (bottom panel) were similar, the median distance of label for pan Shank (middle panel) was larger than those for CaMKII and Shank 2. Since the pan Shank antibody recognized all 3 members of the Shank family (Shank 1, 2 and 3), this difference in laminar distribution between label for pan Shank and Shank 2 could be accounted for by differences in distribution attributed to Shank 1 and Shank 3 [12, 13]. These histograms clearly illustrated that upon high K^+ treatment, the bulk (70-80%) of PSD-associated labels for CaMKII and Shank were located in the PSD pallium.
