# Supplementary material for: Activity-dependent redistribution of CaMKII in the postsynaptic compartment of hippocampal neurons
Source: Mol Brain. 2020 Apr 1;13:53. doi: 10.1186/s13041-020-00594-5 (PMC7110642; doi:10.1186/s13041-020-00594-5)
Supplement: Supplementary file 10 — Additional file 10. [file 13041_2020_594_MOESM10_ESM.pdf]

Additional File 10. Mean and median distance values (nm) of label for CaMKII and Shanks from postsynaptic membrane upon depolarization by high K^+.

	CaMKII		Pan Shank		Shank 2	
**2' K^+**	**Mean± SEM**	**median**	**Mean± SEM**	**median**	**Mean± SEM**	**median**
**Exp 1**	62.4±1.7 (270) [6.7-120]	56.7	62.0±1.8 (230) [6.7-120]	60.0		
**Exp 2**	52.5±1.5 (283) [10-120]	46.7	58.6±1.4 (304) [6.7-120]	56.7	54.7±1.3 (345) [6.7-120]	50
**Exp 3**	56.9±1.9 (210) [6.7-120]	53.3	62.6±1.5 (267) [6.7-120]	60	56.1±1.5 (201) [6.7-113]	53.3
**Mean± SEM**		**52.2±2.9**		**58.9±1.1**		**51.7±1.7**

(n=number of particles measured)

[range: minimum and maximum values in nm]

Statistical analyses by Wilcoxon test for medians within each experiment:

Exp 1: not significant, CaMKII vs. pan Shank.

Exp 2: $P<0.005$, CaMKII vs. pan Shank; not significant, CaMKII vs. Shank 2.

$P<0.05$, pan Shank cs Shank 2.

Exp 3: $P<0.05$, CaMKII vs. pan Shank; not significant, CaMKII vs. Shank 2.

$P<0.01$, pan Shank cs Shank 2.
